# Nucleic Acid Template and the Risk of a PCR-Induced HIV-1 Drug Resistance Mutation

**DOI:** 10.1371/journal.pone.0010992

**Published:** 2010-06-07

**Authors:** Vici Varghese, Elijah Wang, Farbod Babrzadeh, Michael H. Bachmann, Rajin Shahriar, Tommy Liu, Svetlana Jean M. Mappala, Baback Gharizadeh, W. Jeffrey Fessel, David Katzenstein, Seble Kassaye, Robert W. Shafer

**Affiliations:** 1 Department of Medicine, Stanford University School of Medicine, Stanford, California, United States of America; 2 Stanford Genome Technology Center, Stanford University School of Medicine, Stanford, California, United States of America; 3 Department of Pediatrics, Stanford University School of Medicine, Stanford, California, United States of America; 4 Clinical Trials Unit, Kaiser-Permanente Medical Care Program-Northern California, San Francisco, California, United States of America; University of California San Francisco, United States of America

## Abstract

**Background:**

The HIV-1 nucleoside RT inhibitor (NRTI)-resistance mutation, K65R confers intermediate to high-level resistance to the NRTIs abacavir, didanosine, emtricitabine, lamivudine, and tenofovir; and low-level resistance to stavudine. Several lines of evidence suggest that K65R is more common in HIV-1 subtype C than subtype B viruses.

**Methods and Findings:**

We performed ultra-deep pyrosequencing (UDPS) and clonal dideoxynucleotide sequencing of plasma virus samples to assess the prevalence of minority K65R variants in subtype B and C viruses from untreated individuals. Although UDPS of plasma samples from 18 subtype C and 27 subtype B viruses showed that a higher proportion of subtype C viruses contain K65R (1.04% vs. 0.25%; p<0.001), limiting dilution clonal sequencing failed to corroborate its presence in two of the samples in which K65R was present in >1.5% of UDPS reads. We therefore performed UDPS on clones and site-directed mutants containing subtype B- and C-specific patterns of silent mutations in the conserved KKK motif encompassing RT codons 64 to 66 and found that subtype-specific nucleotide differences were responsible for increased PCR-induced K65R mutation in subtype C viruses.

**Conclusions:**

This study shows that the RT KKK nucleotide template in subtype C viruses can lead to the spurious detection of K65R by highly sensitive PCR-dependent sequencing techniques. However, the study is also consistent with the subtype C nucleotide template being inherently responsible for increased polymerization-induced K65R mutations *in vivo*.

## Introduction

The HIV-1 reverse transcriptase (RT) mutation K65R is one of the most important HIV-1 drug resistance mutations. It confers intermediate to high-level resistance to the nucleoside reverse transcriptase (RT) inhibitors (NRTIs) abacavir, didanosine, emtricitabine, lamivudine, and tenofovir, as well as low-level resistance to stavudine [1], [2], [3]. Three lines of evidence suggest that K65R may be more likely to emerge in subtype C than subtype B viruses from individuals with virologic failure: (i) K65R has been reported to emerge more rapidly during in vitro passage of subtype C than in subtype B viruses in the presence of tenofovir [4]. (ii) Observational studies have suggested that K65R emerges in a higher proportion of individuals infected with subtype C who develop virologic failure following combination antiretroviral (ARV) therapy than in those infected with subtype B viruses [5], [6]. (iii) Two laboratories have described preferential RT pausing at the nucleotide position responsible for the AAG-to-AGG K65R mutation during positive-strand DNA synthesis of a subtype C, but not a subtype B, nucleotide template [7], [8], [9]

We performed ultra-deep pyrosequencing (UDPS; 454 Life Sciences/Roche, Branford, CT) to ascertain whether K65R can be detected in minority HIV-1 variants from ARV-naïve individuals infected with subtype C viruses. We also performed UDPS on site-directed mutants and clonal dideoxynucleotide (Sanger) sequencing to assess the influence of PCR-induced mutation in RT genes in the highly conserved KKK RT motif spanning codons 64 to 66 in subtype B and C viruses.

## Methods

### Database analysis of patterns of silent mutations at RT codons 64 to 66 in subtype B and C viruses

We used the HIV Drug Resistance Database [10] to identify patterns of silent mutations at RT codons 63 to 66 in subtype B and C viruses. Because codon 63 — ATA, or isoleucine — is nearly invariant, we examined the nine nucleotides of the wildtype KKK motif in viruses obtained from untreated individuals and of the mutant KRK motif (i.e. with the mutation K65R) in viruses from NRTI-treated individuals. Because direct PCR sequences are less likely than sequences of molecular clones to have PCR errors [11] and because plasma HIV-1 genomes are more likely than proviral DNA genomes to encode viable viruses [12] we analyzed only direct PCR sequences obtained from plasma HIV-1 isolates.

### Clinical HIV-1 samples

We sequenced HIV-1 RT genes from 18 ARV-naïve subtype C-infected individuals whose plasma samples contained HIV-1 RNA levels >4.5 log copies/ml and from which we were able to extract more than 100 distinct virus templates. Sixteen of the samples were obtained between 1999 and 2008 from individuals in the U.S.; two were obtained in 1996 from individuals in Zimbabwe. A collection of 27 virus-load-matched subtype B plasma samples from ARV-naïve individuals were used as controls. We have obtained ethical approval from the Stanford University Institutional Review Board. Because the research involved samples that had already been collected and because the samples were not linked to protected health information, the Institutional Approval included a waiver of informed consent.

### Plasmid clones with subtype B and C nucleotide patterns at the conserved KKK motif

Four types of plasmid clones were created: (i): Four PCR-amplified RT fragments from clinical samples encompassing codons 1 to 127 with the two most common subtype B (AAG-AAA-AAA, AAG-AAA-AAG) and C (AAA-AAG-AAG, AAA-AAG-AAA) KKK patterns were cloned into the pCR2.1 vector (Invitrogen, Carlsbad, CA). (ii) Four PCR-amplified subtype B RT fragments from clinical samples encompassing codons 1 to 127 with the two most common subtype C nucleotide patterns were cloned into the pCR2.1 vector. The large number of subtype B plasma samples available to us allowed us to identify these rare subtype B viruses with subtype C KKK nucleotide patterns. (iii) Site-directed mutants (QuickChange XL Site Directed Mutagenesis Kit, Stratagene, La Jolla, CA) containing the two most common subtype C nucleotide patterns in the subtype B pNL43 clone. (iv) Site-directed mutants containing the two most common subtype B nucleotide patterns in one of the subtype C pCR2.1 clones described above.

### Ultradeep pyrosequencing

Plasma samples were ultracentrifuged and submitted for RNA extraction and reverse transcription using the Roche Amplicor RNA extraction kit and SuperScriptIII RT enzyme (Invitrogen, Carlsbad, CA). The resulting cDNA was submitted for limiting dilution PCR to ensure the presence of >100 distinct cDNA templates. Nested PCRs were then used to amplify RT codons 1 to 127 using both the *Taq-Pwo* enzyme mix (Expand High Fidelity^PLUS^ PCR System, Roche Diagnostics) and the high fidelity polymerase *PfuUltra* II Fusion (Stratagene, La Jolla, CA). Each of the primers used for PCR amplification, including one previously described set of primers and one set optimized for subtype C viruses, is shown in supplementary Table S1. Sequencing was bidirectional with approximately 57% of reads generated by the forward (5′) primers and 43% of reads generated by the reverse (3′) primers. The KKK motif was approximately 180 nucleotides downstream from the start of the sequence generated by the 5′ primers and 180 nucleotides downstream from the start of the sequence generated by the 3′ primers.

Plasmid prep DNA was diluted to approximately 100 distinct DNA templates and PCR amplified using *Taq-Pwo* enzyme mix (Expand High Fidelity^PLUS^ PCR System, Roche Diagnostics) and the high-fidelity enzyme *PfuUltra* II Fusion (Stratagene, La Jolla, CA). *PfuUltra* II Fusion was not used for clinical samples because its amplification efficiency on variable virus templates is often poor [13]. However, it was possible to use *PfuUltra* II Fusion to amplify the plasmid clones because the primers hybridized perfectly to the plasmid template.

PCR products were purified by AMPure kit (Agencourt Biosciences, Beverly, MA), quantified using Quant-iT Picogreen (Invitrogen, Carlsbad, CA), and pooled at equimolar concentrations. Clonal amplification on beads (emulsion PCR) was performed using reagents (emPCR kits II and III; 454 Life Sciences, Branford, CT) that enable bidirectional sequencing. DNA-containing beads were recovered and UDPS was performed on a Genome Sequencer FLX (454 Life Sciences, Branford, CT); each sample pool was loaded in one region of a 70 mm×75 mm PicoTiter plate (454 Life Sciences, Branford, CT) fitted with a four-lane gasket. For 16 of the 18 subtype C samples, UDPS was performed in duplicate on different PicoTiter plates. For these 16 samples, the text reports the mean of the duplicate test results. The coefficient of variation between the two runs was calculated to assess the reproducibility of UDPS on these samples.

### Dideoxynucleoside (Sanger) sequencing

Direct PCR Sanger sequencing encompassing protease and RT codons 1 to 240 was performed on all plasma samples prior to the start of the study using a previously described method [14]. Sanger sequencing of molecular and limiting dilution clones derived from two clinical plasma samples was performed to verify UDPS results. Molecular cloning involved ligating PCR amplicons encompassing codons 1 to 127 into a TA cloning vector (pCR2.1), transforming One Shot® TOP 10F′ Chemically Competent *E.coli* (Invitrogen, Carlsbad, CA), and sequencing plasmid preps generated from individual colonies.

Limiting dilution (LD) clonal sequencing involved subjecting serially diluted cDNA to PCR amplification and sequencing those products resulting from dilutions at which no more than one in three PCR reactions were positive. Sequences lacking electrophoretic mixtures were considered clones and those with electrophoretic mixtures were considered oligoclonal. The thermostable DNA polymerase enzyme mix *Taq-Pwo* (Expand High Fidelity^PLUS^ PCR System) was used for amplification of both molecular and limiting dilution clones. Whereas molecular clones frequently contain detectable PCR errors, limiting dilution clones rarely do because even an error occurring during the first cycle of PCR amplification will rarely be apparent in the final sequence.

## Results

### Database analysis of patterns of silent mutations at RT codons 64 to 66 in subtype B and C viruses

Table 1 compares the variation at the silent third nucleotide position at codons 64 to 66 in HIV-1 subtype B and C viruses. Viruses with the wildtype KKK motif were obtained from NRTI-naïve individuals and viruses with the K65R mutation from NRTI-treated individuals. In the untreated wildtype samples, the two most common variants in subtype B viruses differed from the two most common subtype C variants. The two most common subtype B variants accounted for 97% of the eight possible KKK-encoding nucleotide patterns; six other variants accounted for the remaining 3% of patterns. The two most common subtype C variants accounted for 98% of the eight possible KKK-encoding nucleotide patterns; six variants accounted for the remaining 2% of patterns.

**Table 1:** Comparison of Nucleotide Patterns in Subtype B and Subtype C HIV-1 Reverse Transcriptase Codons 64, 65 and 66 in the HIV Drug Resistance Database.

	NRTI-Naïve Individuals with K65K				NRTI-Treated Individuals with K65R			
	64K	65K	66K	% (n = 7423)	64K	65R	66K	% (n = 365)
	AAG	AAA	AAA	82.73	AAG	AGA	AAA	78.63
	AAG	AAA	AAG	14.25	AAG	AGA	AAG	17.26
	AAG	AAG	AAA	1.90	AAG	AGG	AAA	1.37
	AAA	AAG	AAA	0.70	AAA	AGG	AAA	1.10
	AAA	AAG	AAG	0.28	AAA	AGG	AAG	0.27
	AAG	AAG	AAG	0.08	-	-	-	-
	AAA	AAA	AAA	0.03	AAA	AGA	AAA	1.10
	AAA	AAA	AAG	0.03	AAA	AGA	AAG	0.27

	64K	65K	66K	% (n = 2048)	64K	65R	66K	% (n = 46)
	AAA	AAG	AAG	88.48	AAA	AGG	AAG	91.30
	AAA	AAG	AAA	9.47	AAA	AGG	AAA	8.70
	AAG	AAG	AAG	0.88				
	AAG	AAA	AAG	0.34				
	AAG	AAA	AAA	0.29				
	AAA	AAA	AAG	0.24				
	AAG	AAG	AAA	0.24				
	AAA	AAA	AAA	0.05				

The nucleotide patterns encoding KRK in the subtype B and C isolates from NRTI-treated individuals occurred in proportions similar to the KKK-encoding nucleotide patterns but had a G at the second position of codon 65. Forty individuals had a pre-therapy KKK sample and a post-therapy KRK sample. All but two of the post-therapy samples were identical to the pre-therapy sample except for the presence of an A to G substitution at the second position of codon 65.

### UDPS of subtype B and C plasma virus samples from ARV-naïve individuals

**Figure 1:**
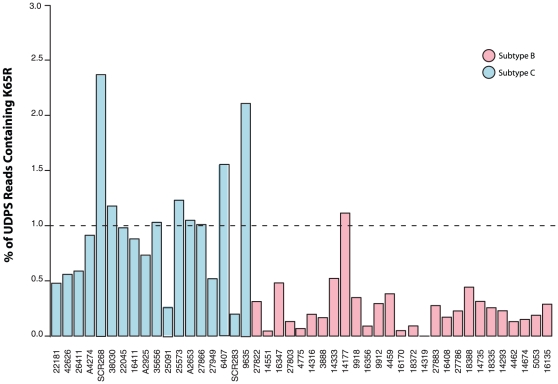
Comparison of Ultra-Deep Pyrosequencing (UDPS) Results of Subtype B and Subtype C Clinical HIV-1 Samples. Proportion of UDPS reads containing K65R in 18 subtype C and 27 subtype B viruses from anti retroviral-naïve individuals. For 16 of the 18 subtype C samples, UDPS was performed twice. The figure shows the mean proportion of K65R for the duplicate runs. The median proportion of K65R reads per sample were 0.25% for subtype B and 1.04% for subtype C.

Figure 1 shows that UDPS detected K65R in a higher proportion of sequence reads of subtype C plasma samples than in subtype B plasma samples (1.04% vs. 0.25%; p<0.001 Wilcoxon Rank Sum Test). K65R was detected in at least 1.0% of reads in 8/18 (44%) subtype C samples and in 1/27 (3.7%) subtype B samples (p = 0.01; Fisher's Exact Test).

Table 2 shows the dominant wildtype KKK nucleotide patterns in the UDPS reads of the 18 subtype C and 27 subtype B viruses and the KRK nucleotide patterns for those samples in which K65R was present in at least 1.0% of UDPS reads. The two most common subtype B wildtype KKK nucleotide patterns matched the two most commonly reported subtype B nucleotide patterns. The most common dominant subtype C wildtype KKK nucleotide pattern matched the most commonly reported subtype C nucleotide pattern.

**Table 2:** Dominant Intra-Individual Pattern of Nucleotides Coding for K64, K65 and K66 in 27 Subtype B and 18 Subtype C Isolates from Untreated Individuals: Association with presence of K65R as Determined by Ultra-Deep Pyrosequencing (UDPS)*.

Subtype	KKK Motif (wildtype K65K)	No. Isolates	K65R (≥1.0%)	KRK Sequence (K65R)
B	AAG AAG AAA†	1	1 (1.1%)	AAA A**G**A AAA
	AAA AAG AAG	0	0	Not applicable
	AAG AAA AAA	22	0	Not applicable
	AAG AAA AAG	4	0	Not applicable
	AAG AAG AAG	0	0	Not applicable
C	**AAA AAG AAA**	**1**	**1 (2.1%)**	AAA A**G**G AAA
	**AAA AAG AAG**	**12**	**7 (1.0% to 2.3%)**	AAA A**G**G AAG
	AAG AAA AAA	4	0	Not applicable
	AAG AAA AAG	0	0	Not applicable
	AAG AAG AAG	1	0	Not applicable

*The column “KKK Motif” indicates the Sanger sequence of the codons 65 to 67 in the plasma virus samples. The column “No. Isolates” indicates the number of individuals with the Sanger sequence shown in the “KKK Motif column”. The column “K65R (≥1.0%)” indicates the number of samples for which ≥1.0% of the reads had K65R. The range in proportions is shown in parentheses. The proportion of reads with K65R was determined by dividing the number of reads with K65R by the total number of sequencing reads encompassing codon 65.

†This sample had a complex mixture of KKK nucleotide variants including AAA-AAG-AAA (the second-most common subtype C pattern) in 38% of reads, AAG-AAA-AAA (the most common subtype B pattern) in 7% of reads, and the uncommon pattern AAG-AAG-AAA in 50% of reads.

Among the subtype B samples, K65R was detected in more than 1.0% of reads in a sample with a complex mixture of KKK nucleotide variants including AAA-AAG-AAA (the second-most common subtype C pattern) in 38% of reads, AAG-AAA-AAA (the most common subtype B pattern) in 7% of reads, and an uncommon pattern AAG-AAG-AAA in 50% of reads. Despite the fact that the K65R reads in this subtype B sample (1.1% of all reads) were encoded by AAA-AGA-AAA, there were no reads with the closest wildtype variant, AAA-AAA-AAA.

Among the subtype C samples, K65R was detected in at least 1.0% of reads in eight samples, including 7 of 11 with the most common subtype C pattern and the one sample with the second-most common subtype C pattern. Of the four subtype C samples with the most commonly reported subtype B KKK nucleotide variant (AAG-AAA-AAA), none had K65R in 1.0% or more of reads.

The coefficient of variation for the duplicate UDPS runs on the subtype C sequences was 0.23. For eight of the 16 repeated subtype C samples, the proportion of reads with K65R was >1.0% in each of the duplicate runs and for seven of the 16 repeated subtype C samples, the proportion of reads with K65R was <1.0% on both runs. The median proportion of reads with K65R was similar in the forward (0.93%) and reverse (1.2%) UDPS reads.

### Clonal analysis of two subtype C viruses for which more than 2.0% of UPDS reads contained K65R

To determine whether the high frequency of K65R reads in subtype C viruses reflected a natural occurrence of this mutation at low levels in subtype-C-infected individuals or a technical artifact of either PCR or UDPS, we performed clonal dideoxynucleotide sequencing on the two subtype C viruses with the highest proportions of K65R reads. Table 3 shows that, although 133 of 5186 UDPS reads of sample SCR268 had K65R, none of 265 limiting dilution clones of the same sample had the mutation (p = 0.001; Fisher's Exact Test). Similarly, although 57 of 3613 UDPS reads of sample 9635 had K65R, none of the 254 limiting dilution clones we sequenced of this sample had the mutation (p = 0.02, Fisher's Exact Test). In contrast, there was no statistically significant difference in the number of UDPS vs. molecular clones for sample SCR268 (6 of 278 vs. 133 of 5186; p = 0.8) or 9635 (3 of 278 vs. 57 of 3613; p = 0.8). Although limiting dilution sequencing did not detect K65R, it did detect each of the other 33 nucleotide and three amino acid mutations that were present in more than 1.0% of UDPS reads in these two samples. Taken together, the limiting dilution and molecular cloning sequencing results strongly suggest that PCR error was responsible for the subsequent frequent false positive detection of K65R by UDPS of subtype C samples.

**Table 3:** Comparison of Ultra-Deep Pyrosequencing (UDPS) with Limiting Dilution Clonal Sequence on the Two Samples with the Highest Proportion of K65R.

Sample	UDPS†			Limiting Dilution Clones			p Value*
	UDPS reads	K65R reads	% K65R reads	Total clones	K65R clones	% K65R clones	
SCR268	**5186**	**133**	**2.6%**	265	0	0	p = 0.001
9635	**3613**	**57**	**1.6%**	254	0	0	p = 0.02

*Fisher's Exact Test.

†Total reads from two separate UDPS runs. The proportion of reads with K65R was 2.9% for one UDPS run and 1.9% for the second UDPS run for SCR268. Overall, 2.6% of total reads contained K65R. The proportion of reads with K65R was 2.7% for one UDPS run and 1.2% for the second UDPS run for 9635. Overall, 1.6% of total reads contained K65R.

### UDPS KKK sequences of site-directed mutants and plasmid clones

Table 4 shows that a median of 1.2% of 454 UDPS reads (range: 0.7%–1.3%) of the eight clones with the two most common subtype C KKK patterns had K65R. In contrast, the four clones with the two most common subtype B KKK patterns had K65R in a median of 0.15% of reads (range 0.1%–0.3%; P = 0.007; Wilcoxan Rank Sum Test). The proportion of UDPS reads containing K65R appeared to be entirely dependant on the KKK pattern in the sample. The subtype from which the surrounding viral sequence was derived did not significantly influence the number of reads with K65R. For plasmid clones with a subtype C nucleotide pattern, UDPS detected K65R in a mean 1.1% of reads (range 0.7–1.3). In contrast, for plasmid clones with a subtype B nucleotide pattern, UDPS detected K65R in a mean of 0.2% reads (range 0.1–0.3).

**Table 4:** Ultradeep-Pyrosequencing of PCR-Induced Mutations at Codon 65 in Plasmid Clones with Different KKK Nucleotide Patterns in Subtype B and Subtype C Genetic Contexts.

Vector	Insert	Amplicon Subtype	KKK Motif Subtype	KKK Nucleotide Sequence	Reads at Codon 65	% of K65R Reads
pCR2.1	SDM	C	C1	AAA-AAG-AAG	7308	0.7
NL43	SDM	B	C1	AAA-AAG-AAG	4726	0.8*
pCR2.1	Amplicon	B	C1	AAA AAG AAG	14742	1.3
pCR2.1	Amplicon	B	C1	AAA AAG AAG	15255	1.1
NL43	SDM	B	C2	AAA-AAG-AAA	6175	1.2*
pCR2.1	Amplicon	C	C2	AAA-AAG-AAA	2881	1.1*
pCR2.1	Amplicon	B	C2	AAA AAG AAA	12424	1.2
pCR2.1	Amplicon	B	C2	AAA AAG AAA	14216	1.3
NL43	Amplicon	B	B1	AAG AAA AAA	6407	0.1*
pCR2.1	SDM	C	B1	AAG AAA AAA	6755	0.2*
NL43	SDM	B	B2	AAG AAA AAG	4789	0.1
pCR2.1	SDM	C	B2	AAG AAA AAG	6270	0.3*

SDM, Site directed mutant; Amplicon subtype, subtype of RT codons 1–127; Underlined bases indicate bases changed by SDM; Reads at codon 65, average number of reads or sequence coverage at codons 64 to 66.

*Samples for which *PfuUltra* II Fusion was used in addition to *Taq-Pwo* polymerase. The % of K65R reads with *PfuUltra* II Fusion ranged from 0.02 to 0.05 for the three samples with the subtype C nucleotide patterns and 0 to 0.09 for the three samples with B nucleotide patterns.

For clones with three subtype C KKK patterns, cDNA was amplified with *PfuUltra* II Fusion, as well as *Taq/Pwo* polymerase (Expand High Fidelity^PLUS^ PCR System). The proportion of K65R clones was 16-fold to 80-fold lower in the samples amplified using *PfuUltra* II Fusion than in the samples amplified with *Taq/Pwo* (Table 4 footnote).

## Discussion

Inter-subtype nucleotide differences have been shown to influence the likelihood of certain HIV-1 drug resistance mutations. For example, subtype C viruses are significantly more likely than subtype B viruses to develop the non-nucleoside RT-inhibitor-resistance mutation V106M because this mutation requires a single substitution for subtype C viruses (GTG→ATG) but two substitutions for subtype B viruses (GTA→ATG ) [15], [16]. However, such a mechanism cannot explain a preference for K65R in subtype C viruses because a change from K to R requires a single substitution regardless of the underlying nucleotide template (AAA→AGA or AAG→AGG).

Although UDPS detected K65R in a median of 1.0% of sequence reads of subtype C plasma samples, Sanger sequencing of limiting dilution and molecular clones from the two samples with the highest proportions of K65R strongly suggested that the UDPS data resulted from PCR error. Molecular clones are subject to PCR errors because a PCR error occurring during an early round of amplification will result in large numbers of clones containing a sequencing error. Limiting dilution sequencing circumvents this problem because even if an error occurs in the first cycle to PCR, it will be present in no more than 25% of the bases at that position in the amplified DNA and would not result in an erroneous base call when the amplified DNA is sequenced [11].

A second set of UDPS experiments with plasmid clones containing the two most common subtype B (AAG-AAA-AAA and AAG-AAA-AAG) and C (AAA-AAG-AAG and AAA-AAG-AAA) nucleotide templates demonstrates unequivocally that the sequence of this template alone was sufficient to explain the higher PCR error rate with subtype C nucleotide templates. These experiments also confirm that it was PCR error, rather than pyrosequencing that accounts for the spurious finding of K65R. Indeed, K65R was not detected by UDPS when the high fidelity PCR enzyme *PfuUltra* II Fusion was used. The fact that codon 63 has an invariant ATA sequence is consistent with the idea that the increased likelihood of K65R is due to the stretch of six consecutive T's encountered during positive-strand DNA synthesis of subtype C (but not subtype B) templates [7].

Our study has two important implications. First, it shows that the RT KKK nucleotide template in subtype C viruses can lead to the spurious detection of K65R by highly sensitive PCR-dependent sequencing techniques such as UDPS or deep molecular clonal sequencing. The risk of such “false-positive” results can be reduced by using PCR enzymes with higher fidelity and by decreasing the need for PCR during upstream processing. Second, our finding that the likelihood of identifying K65R varies with viral subtype is consistent with the work of Coutsinos et al [7]. Thus, even though naturally occurring K65R variants do not appear to be present in ARV-naïve individuals at levels detectable by currently available deep sequencing technologies, we cannot exclude the possibility that the steady state levels of such variants are higher in subtype C compared with subtype B virus isolates.

## Supporting Information

Table S1 PCR Amplification Strategy for Ultra-deep Pyrosequencing of Subtype C Samples.
(0.03 MB DOC)
