# Supplementary material for: Nucleic Acid Template and the Risk of a PCR-Induced HIV-1 Drug Resistance Mutation
Source: PLoS One. 2010 Jun 7;5(6):e10992. doi: 10.1371/journal.pone.0010992 (PMC2881873; doi:10.1371/journal.pone.0010992)
Supplement: Table S1 — PCR Amplification Strategy for Ultra-deep Pyrosequencing of Subtype C Samples. (0.03 MB DOC) [file pone.0010992.s001.doc]

| **Supplementary Table 1. PCR Amplification Strategy for Ultra-deep Pyrosequencing of Subtype C Samples** | | | |
| --- | --- | --- | --- |
| Target-specific primer sequence | Direction | Position on HXB2 genome | PR*/RT codon position |
| CCTCARATCACTCTTTGGC | Sense | 2253→2271 | PR1-PR7 |
| YTTGGGCCATCCATTCCTGG | Antisense | 2608→2589 | RT14-RT20 |
| TGCACAYTAAATTTTCCAATTAG | Sense | 2535→2557 | PR95-RT3 |
| ACTAGGTATGGTGAATGCAG | Antisense | 2951→2932 | RT128-RT134 |
| CTRGATGTGGGRGATGCA | Sense | 2874→2891 | RT109-114 |
| TGTWTWGGCTGTACTGTCC | Antisense | 3284→3265 | RT239-RT245 |
| Each primer also comprised one of two adaptor sequences from the 454 Life Sciences platform (GCCTCCCTCGCGCCATCAG and GCCTCCCTCGCGCCATCAG) and one 4-nucleotide barcode (ACTT, ATCA, TCTG, TACT, CTCT or CTCA) in the following 5’ to 3’ orientation: 454 adaptor-barcode-primer. Thus, a total of 36 (3 X 2 X 6) primers were used to encompass protease and first 238 amino acids of RT and to enable pooling of 6 samples. *PR = protease | | | |
